# 8-Prenylgenistein, a prenylated genistein derivative, exerted tissue selective osteoprotective effects in ovariectomized mice

**DOI:** 10.18632/oncotarget.24823

**Published:** 2018-03-19

**Authors:** Yan Zhang, Li-Ping Zhou, Xiao-Li Li, Yong-Jian Zhao, Ming-Xian Ho, Zuo-Cheng Qiu, Dong-Feng Zhao, Daniel Kam-Wah Mok, Qi Shi, Yong-Jun Wang, Man-Sau Wong

**Affiliations:** ^1^ Spine Research Institute, Longhua Hospital, Shanghai University of Traditional Chinese Medicine, Shanghai, PRC; ^2^ State Key Laboratory of Chinese Medicine and Molecular Pharmacology (Incubation), The Hong Kong Polytechnic University Shenzhen Research Institute, Shenzhen, PRC; ^3^ Key Laboratory of Theory and Therapy of Muscles and Bones of Ministry of Education, Shanghai, PRC; ^4^ Department of Applied Biology and Chemical Technology, The Hong Kong Polytechnic University, Hung Hom, Kowloon, Hong Kong, PRC; ^5^ School of Medical Instrument and Food Engineering, University of Shanghai for Science and Technology, Shanghai, PRC; ^6^ Institute of Traditional Chinese Medicine and Natural Products, College of Pharmacy, Jinan University, Guangzhou, PRC; ^7^ School of Rehabilitation Science, Shanghai University of Traditional Chinese Medicine, Shanghai, PRC

**Keywords:** genistein, 8-prenylgenistein, bone, estrogenic effects, uterus

## Abstract

Our previous study reported that the *in vitro* osteogenic effects of 8-prenylgenistein (8PG) were more potent than its parent compound genistein. This study aimed to evaluate the osteoprotective effects of 8PG in ovariectomized (OVX) mice as well as to characterize its estrogenic effects in uterus. Mature OVX mice were treated with phytoestrogen-free diet containing 8PG or genistein. Trabecular bone mass and most of the micro-structural parameters were ameliorated at the distal femoral metaphysis in OVX mice upon treatment with genistein and both doses of 8PG. The beneficial effects of 8PG on trabecular bone were confirmed by safranin O and ABHO staining. 8PG markedly inhibited the ovariectomy-induced mRNA expressions of RANKL/OPG, ALP, COL, OCN, cathepsin K and ER-α in bone. In contrast, genistein further increased the ovariectomy-induced ER-α expression in bone. The uterus index was increased in genistein-treated group. Genistein up-regulated the expression of ER-α and PR, while 8PG significantly down-regulated the ER-α and C3 expression in uterus of OVX mice. Moreover, genistein, but not 8PG, increased expressions of ER-α, PCNA and C3 in Ishikawa cell. This study suggested that 8PG improved trabecular bone properties in OVX mice without exerting uterotrophic effects and its estrogenic actions were distinct from those of genistein.

## INTRODUCTION

Soybean-based foods have been regarded as healthy food choices as a result of the evidence generated from epidemiological studies that populations consuming large amounts of soybeans have a lower risk of developing chronic diseases, notably osteoporosis [1, 2]. Soy foods contain an array of biologically-active phytochemicals that may confer important health benefits [3]. These compounds include the isoflavones, so-called phytoestrogens, which have received considerable attention because of their estrogen-like properties in certain tissues, including bone [4–6]. However, as the *in vivo* effects of phytoestrogens are similar to estrogens and their actions are mediated through estrogen receptors (ER-α and ER-β), concerns for the safety of using phytoestrogens amongst medical scientists are increasing [7, 8]. These concerns have led to the clinical studies that evaluate the safety of soy isoflavones on reproductive organs in postmenopausal women [8] and infants [9]. Thus, the major focus of phytoestrogens research and bone health is to identify novel estrogenic compounds that exhibit tissue selectivity in reducing menopausal related bone loss without inducing undesirable stimulatory effects in reproductive tissues.

Prenylated flavanones, a unique class of naturally occurring flavonoids, are characterized by the presence of a prenylated side chain in the flavonoid skeleton [10]. This class of compound has recently attracted much attention due to their exhibition of a wide range of interesting pharmacological properties, including estrogenic, anti-inflammatory, anti-viral and anti-oxidative effects, especially their superior activities over those of its parent compound [11]. Emerging evidences showed that the prenylation at different sites produces differential biological responses [11–13] and the presence of 8-prenylation increased the bioactivities of the parent flavonoid compounds, such as 8-prenylnaringenin (8PN) [14, 15] and 8-prenylapigenin [16]. As compared to naringenin, 8PN led to a strong increase in estrogenicity as observed in the abilities to induce transactivation activities of the yeast screen estrogen receptor assay, stably transfected luciferase reporter activities in MVLN cells derived from human breast cancer cells and alkaline phosphatase (ALP) activities of Ishikawa cells [17]. In addition, the estrogenic activities of 8PN *in vitro*, including the ability to induce estrogen responsive reporter activities in yeast and ALP activities in Ishikawa cells, were greater than those of the established phytoestrogens such as coumestrol, genistein and daidzein, and its high estrogenic activity was confirmed *in vivo* as demonstrated by the induction of uterine vascular permeability in mice in response to subcutaneously administration of 8PN [18].

**Figure 1:**
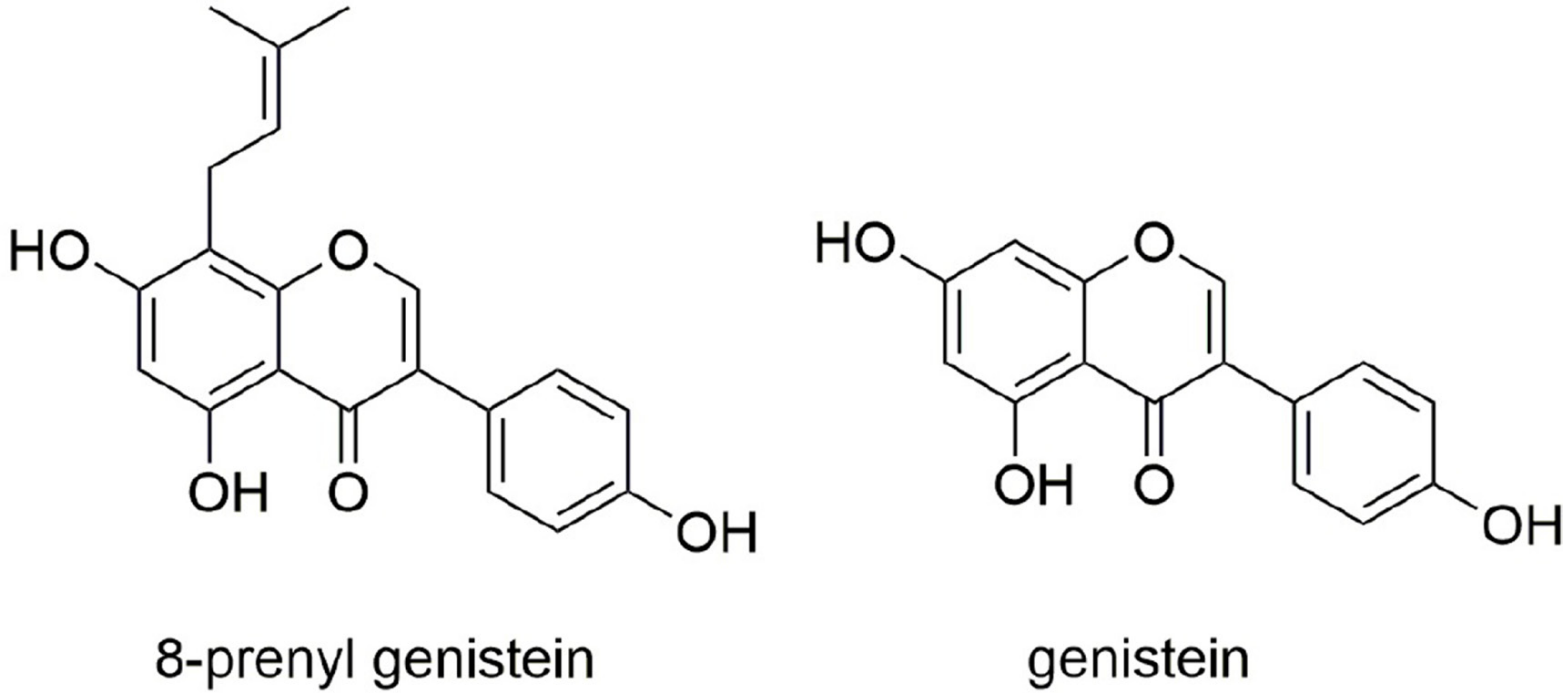
Chemical structure of genistein and 8-prenylgenistein (8PG)

The altered estrogenicity induced by the modification of prenylation appears to play a significant role in contributing to the actions of different flavonoid compounds on bone metabolism. The existence of a prenyl group on C-8 position of icariin has been suggested to be the reason why icariin is more potent than genistein in osteogenic activity [19]. Moreover, the prenyl group on 8PN was also found to play a significant role in contributing to its activities in enhancing bone formation and inhibiting bone resorption *in vitro* [15, 20]. Indeed, 8PN has a more pronounced ability than naringenin in enhancing osteoblast differentiation and mineralization [21]. Our group previously studied the structure-activity relationship of genistein (Figure 1) with prenylation at different position and found that the prenylation at position C-8 of ring A, namely 8-prenylgenistein (8PG, Figure 1), displayed higher potency in stimulating the proliferation, differentiation and mineralization of osteoblasts [22, 23]. The results revealed that 8PG might be the bioactive compound in *Erythrina variegata* L. (EV) that accounted for the bone protective actions in ovariectomy-induced osteoporotic rats [24] and mice [25]. These studies clearly indicated that the prenylation at C-8 position of flavonoid compound is important for inducing osteogenesis. However, the *in vivo* osteoprotective efficacy of 8PG is yet to be determined.

With the discovery of multiple ERs that can mediate the effects of estrogen and the complexity of estrogen signaling, concerns are raised about the selectivity of different endocrine disrupting chemicals (including phytoestrogens) towards estrogen sensitive tissues [26]. It is important to understand how tissue selectivity of estrogenic compounds can be achieved. Thus, to assess the risks and benefits of using 8PG for the management of postmenopausal osteoporosis, the evaluation on its tissue selectivity as well as the elucidation of its molecular actions is warranted. The present study was performed to evaluate the estrogenic effects of 8PG in bone and uterus using ovariectomized mice as *in vivo* model and osteoblastic (MG-63 and MC3T3-E1) cells and human endometrial (Ishikawa) cells as *in vitro* model.

## RESULTS

### Body weight and biochemistries in serum and urine

The effects of genistein and 8PG at low and high dose on serum and urine chemistries and body weight were shown in Table 1. Ovariectomy induced the increase of body weight in mice (*P* < 0.01, vs Sham), and treatment with genistein or 8PG did not affect body weight of OVX mice. Urinary Ca and P levels were significantly increased in mice in response to ovariectomy (*P* < 0.05, vs Sham). Urinary Ca level was significantly reduced by 62% in OVX mice treated with high dose of 8PG (*P* < 0.05, vs OVX). Serum OCN (a bone formation marker, *P* < 0.05) and OPN (a bone resorption marker, *P* < 0.001) levels in OVX group were both increased as compared to those in Sham group, indicating an increase in bone turnover in mice induced by ovariectomy. Genistein and 8PG failed to alter the level of serum OCN in OVX mice. In contrast, both genistein (*P* < 0.05) and 8PG (*P* < 0.001) significantly decreased serum OPN level, which was not statistically different between high dose of 8PG group and Sham group.

**Table 1:** Body weight, uterus index and chemistries in serum and urine

	Sham	OVX	Gen	8PG-L	8PG-H
Body weight (g)	22.1 ± 0.5	24.2 ± 0.3^##^	23.6 ± 0.3	24.7 ± 0.4^##^	24.8 ± 0.4^##^
Uterus index (mg/g)	2.61 ± 0.21	0.47 ± 0.02^###^	0.64 ± 0.03^***^	0.46 ± 0.01^###^	0.48 ± 0.03^###^
Serum Ca (mg/dL)	11.02 ± 0.21	10.68 ± 0.28	10.47 ± 0.43	11.79 ± 0.42	11.08 ± 0.49
Serum P (mg/dL)	6.60 ± 0.48	6.45 ± 0.29	7.08 ± 0.41	6.69 ± 0.20	7.12 ± 0.41
Serum OCN (ng/ml)	22.9 ± 6.6	50.9 ± 6.6^#^	58.5 ± 8.5^##^	52.7 ± 4.2^#^	61.7 ± 5.0^###^
Serum OPN (ng/ml)	12.5 ± 0.6	18.6 ± 0.5^###^	16.5 ± 0.7^###*^	14.9 ± 0.4^***#^	13.1 ± 0.3^***^
Urine Ca/Cr (mg/mg)	0.182 ± 0.053	0.470 ± 0.121^#^	0.213 ± 0.031	0.221 ± 0.062	0.180 ± 0.030^*^
Urine P/Cr (mg/mg)	1.45 ± 0.25	3.95 ± 0.37^##^	4.33 ± 0.61^###^	3.15 ± 0.56	3.25 ± 0.31^#^

Values were expressed as means ± SEM, *n* = 10 in each group. ^#^*P* < 0.05, ^##^*P* < 0.01, ^###^*P* < 0.001, vs. Sham group, ^*^P < 0.05, ^***^P < 0.001, vs. OVX group. Ca, calcium; Cr, creatinine; P, phosphorus; OCN, osteocalcin; OPN, osteopontin.

### Micro CT analysis

**Figure 2:**
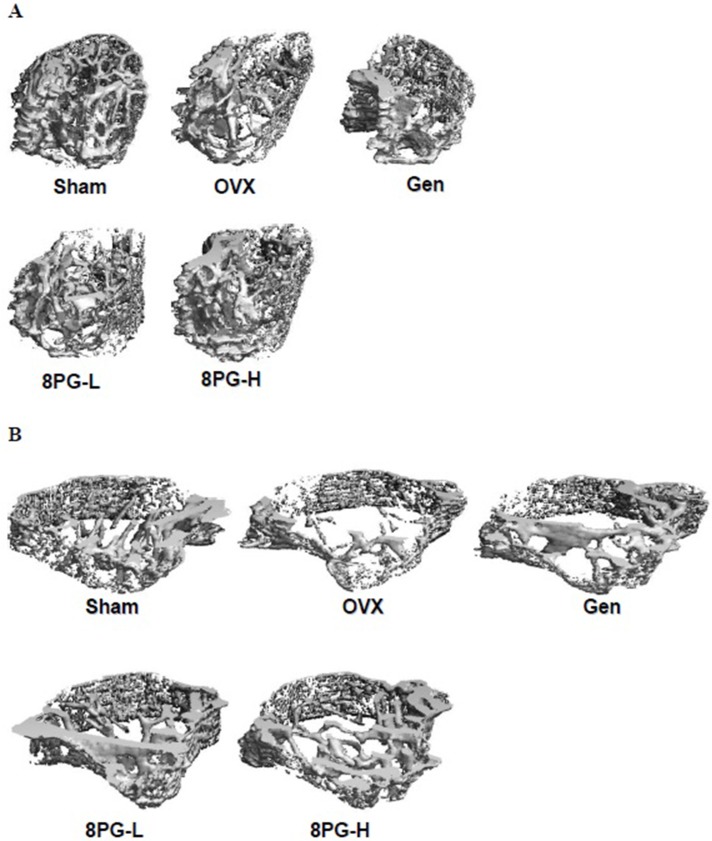
A representative microcomputed tomography 3-dimensional image of the trabecular bone at distal femoral end (**A**) and at proximal tibial head (**B**) in Sham mice and OVX mice treated with vehicle (OVX), genistein (Gen) or low (8PG-L) or high (8PG-H) dose of 8-prenylgenistein for 7 weeks.

The effects of 8PG as compared to genistein on trabecular bone properties of OVX mice were determined at the distal end of femur and the proximal metaphysis of tibia. The profiles of 3D images clearly demonstrated the loss of trabecular bone mass and trabecular bone number as well as the deterioration of cancellous bone structure at the distal end of femur (Figure 2A) and the proximal metaphysis of tibia (Figure 2B) in OVX mice. Such morphological changes for trabecular bone in OVX mice were quantitatively reflected by the micro-CT measurement of bone parameters as shown in Table 2. Ovariectomy significantly induced the decrease in BMD/TV, Conn.D, BV/TV, Tb.N, and the increase in Tb.Sp at proximal tibial metaphysis (Table 2A) and distal femoral end (Table 2B) in mice (*P* < 0.05, vs Sham).

**Table 2:** Effects of 8-prenylgenistein on trabecular bone parameters measured by micro-CT at the proximal metaphysis of tibia (A) and the distal metaphysis of femur (B) in ovariectomized mice

**A**					
**Parameters\Group**	**Sham**	**OVX**	**Gen**	**8PG-L**	**8PG-H**
BMD/TV (mg HA/ccm)	105 ± 7	59 ± 3^##^	67 ± 9	74 ± 6	85 ± 6^*^
Conn.D (1/mm^3^)	360 ± 55	136 ± 31^#^	211 ± 32	197 ± 15	202 ± 18
BV/TV (%)	87.9 ± 5.9	50.7 ± 5.7^##^	67.7 ± 12.8	71.3 ± 11.3	77.0 ± 13.5
DA	1.35 ± 0.04	1.81 ± 0.03^#^	1.60 ± 0.08	1.34 ± 0.05^*^	1.34 ± 0.03^*^
Tb.N (1/mm)	2.7 ± 0.1	1.6 ± 0.1^###^	1.9 ± 0.2	1.9 ± 0.2	1.9 ± 0.2
Tb.Sp μm)	337 ± 17	591 ± 48^##^	498 ± 72	509 ± 55	439 ± 43^*^
Tb.Th (μm)	33.3 ± 1.0	30.8 ± 2.2	33.8 ± 4.9	36.7 ± 2.3	39.8 ± 1.4^**^
**B**					
**Parameters\Group**	**Sham**	**OVX**	**Gen**	**8PG-L**	**8PG-H**
BMD/TV (mg HA/ccm)	149 ± 15	97 ± 7^#^	137 ± 7^*^	141 ± 13^*^	151 ± 8^**^
Conn.D (1/mm^3^)	266 ± 40	135 ± 26^#^	210 ± 40	179 ± 19	192 ± 22
BV/TV (%)	16.6 ± 2.1	10.1 ± 0.6^#^	16.8 ± 0.9^**^	15.9 ± 1.9^*^	19.7 ± 1.2^**^
DA	1.27 ± 0.02	1.34 ± 0.10	1.34 ± 0.05	1.25 ± 0.03	1.30 ± 0.07
Tb.N (1/mm)	3.5 ± 0.2	2.3 ± 0.1^##^	3.0 ± 0.2^*^	3.1 ± 0.2^*^	3.2 ± 0.2^*^
Tb.Sp (μm)	243 ± 17	385 ± 13^###^	273 ± 17^**^	295 ± 27^*^	257 ± 24^**^
Tb.Th (μm)	52.3 ± 2.2	43.5 ± 2.9^#^	54.9 ± 3.8	59.6 ± 3.7^*^	62.4 ± 0.8^**^

Values were expressed as means ± SEM, *n* = 8 in each group. ^#^*P* < 0.05, ^##^*P* < 0.01, ^###^*P* < 0.001, vs. Sham; ^*^P < 0.05, ^**^P < 0.01, vs. OVX. BMD/TV, bone mineral density over total volume; Conn.D, connectivity density; BV/TV, bone volume over total volume; DA, degree of anisotropy; Tb.N, trabecular bone number; Tb.Sp, trabecular bone separation; Tb.Th, trabecular bone thickness.

Genistein appeared to moderately improve the tibial parameters for bone mass and micro-architecture in OVX mice but the changes were not statistically significant (Table 2A). Low dose of 8PG significantly decreased DA (*P* < 0.05), and high dose of 8PG dramatically increased BMD/TV (*P* < 0.05) and Tb. Th (*P* < 0.01) and decreased DA (*P* < 0.05) and Tb. Sp (*P* < 0.05) of proximal metaphysis of tibia in OVX mice (vs OVX, Table 2A). The effects of genistein and two doses of 8PG were more prominent at distal metaphysis of femur in OVX mice (Table 2B). All phytoestrogens treatments could significantly increase BMD/TV and BV/TV, and improve trabecular bone architectural parameters including Tb. N and Tb. Sp at distal femur of OVX mice (*P* < 0.05, vs OVX).

### Bone histomorphology

**Figure 3:**
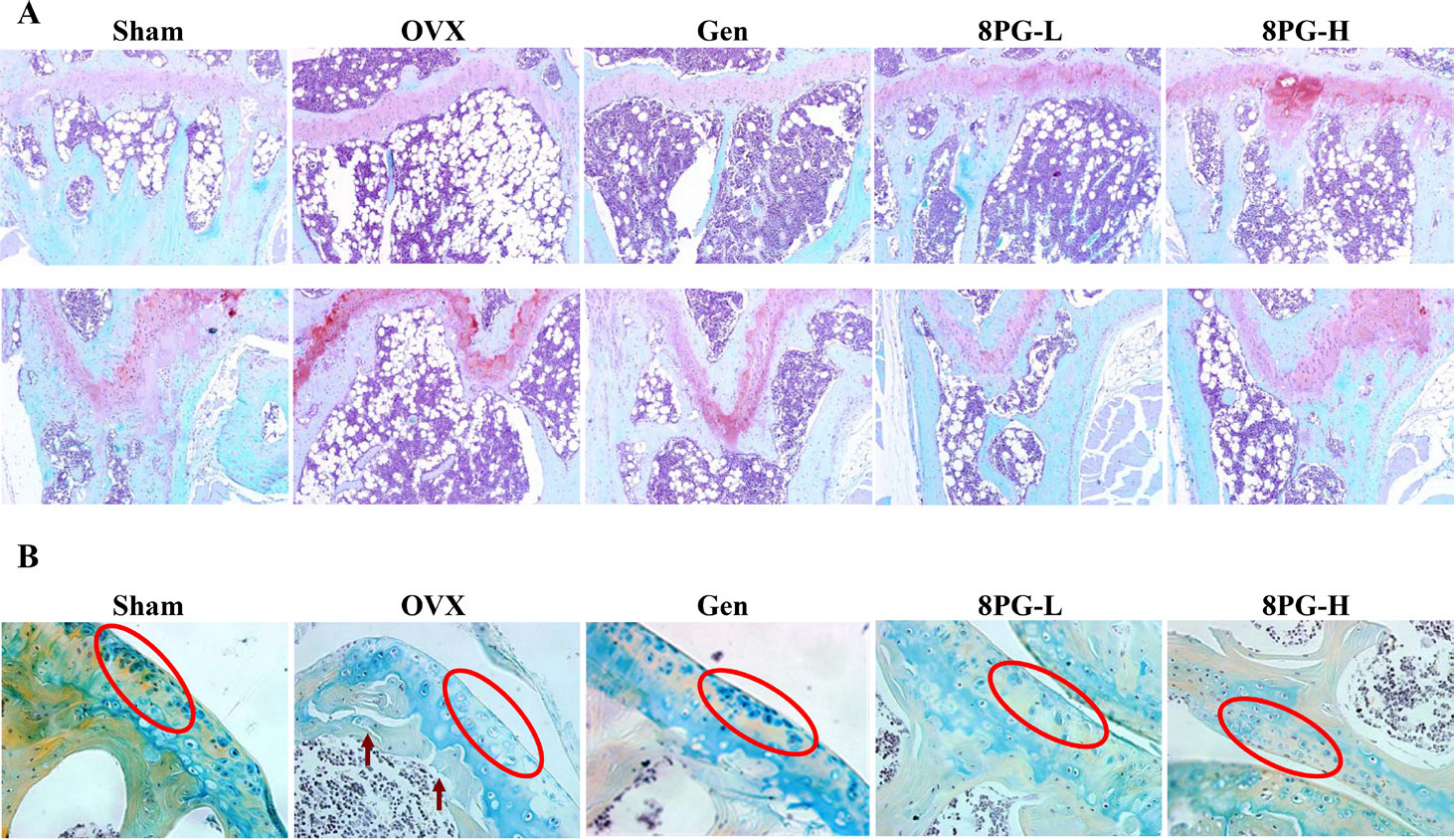
Histological images of proximal tibial metaphysis (**A**, top panel) and distal femoral metaphysis (**A**, bottom panel) measured by Safranin O staining (**A**, Magnification, ×100) and Alcian blue/Hematoxylin & Orange G staining (**B**, Magnification, ×400) in Sham mice and OVX mice treated with vehicle (OVX), genistein (Gen) or low (8PG-L) or high (8PG-H) dose of 8-prenylgenistein for 7 weeks. Red circle, articular chondrocytes; Brown arrow, cleft of articular cartilage.

To confirm the beneficial effects of 8PG on trabecular bone, the local histomorphology was performed on proximal tibial metaphysis and distal femoral end (Figure 3). The safranin O staining (Figure 3A) showed the loss and destruction of woven trabecular bone in OVX mice, and the Alcian blue/Hematoxylin & Orange G (ABHO) staining (Figure 3B) showed the loss of articular chondrocytes (red circle) at the surface of knee joint and the clefts of articular cartilage (brown arrow) at epiphyseal area of proximal tibial metaphysis in OVX mice as compared to those of Sham rats. Treatment with genistein moderately attenuated the ovariectomy-induced pathological changes of trabecular bone at tibia (Figure 3A, top panel) and femur (Figure 3A, bottom panel) in OVX mice. Most importantly, 8PG at both doses increased the connection and the amount of trabecular bone at tibia and femur (Figure 3A) as well as increased the number of chondrocytes and recovered the structure of articular cartilage (Figure 3B) at proximal tibial epiphysis in OVX mice.

### Expression of key regulators for bone metabolism

**Figure 4:**
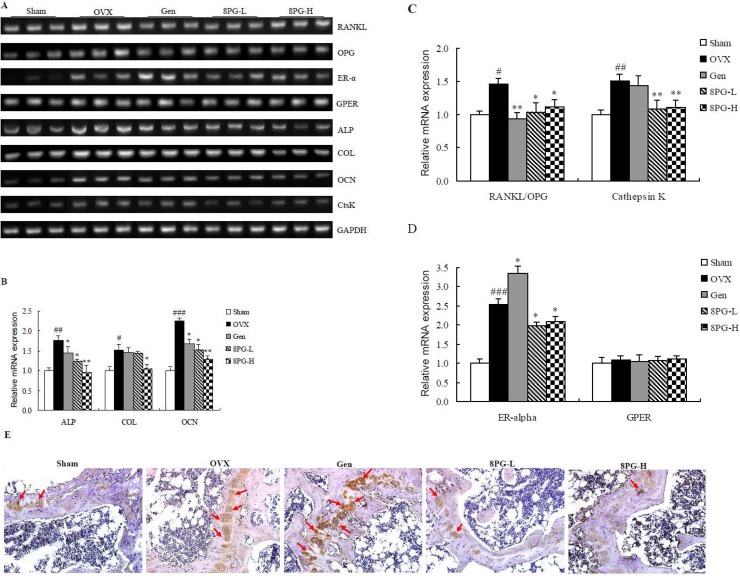
mRNA expression of receptor activator of nuclear factor kappa-B ligand (RANKL), osteoprotegerin (OPG), estrogen receptor alpha (ER-α), G protein-coupled estrogen receptor (GPER), alkaline phosphatase (ALP), type I collagen (COL), osteocalcin (OCN), cathepsin K (CtsK), and protein expression of ER-α in distal femoral end from Sham mice and OVX mice treated with vehicle (OVX), genistein (Gen) or low (8PG-L) or high (8PG-H) dose of 8-prenylgenistein for 7 weeks (**A**) Agarose gel electrophoresis. (**B**–**D**) the quantitative PCR for the mRNA expression levels, which were expressed as a ratio to the expression of GAPDH. (**E**) Immunostaining of ER-α expression with positive signal shown by red arrow (Magnification, ×200). Values were expressed as means ± SEM, *n* = 8. ^#^*P* < 0.05, ^##^*P* < 0.01, ^###^*P* < 0.001, vs. Sham group; ^*^P < 0.05, ^**^P < 0.01, vs. OVX group.

To further elucidate the mechanisms of actions of 8PG in bone, the mRNA expression of osteoblast-specific genes and the osteoclast-specific gene cathepsin K (CtsK) as well as genes involved in estrogenic signaling like estrogen receptor alpha (ER-α) and G protein-coupled estrogen receptor (GPER) in distal femur of mice were measured (Figure 4). The mRNA expression of alkaline phosphatase (ALP) (Figure 4B, *P* < 0.01), type I collagen (COL) (Figure 4B, *P* < 0.05), osteocalcin (OCN) (Figure 4B, *P* < 0.001), the ratio receptor activator of nuclear factor kappa-B ligand (RANKL)/osteoprotegerin (OPG) (Figure 4C, *P* < 0.05), cathepsin K (CtsK) (Figure 4C, *P* < 0.01), and ER-α (Figure 4D, *P* < 0.001) in femur were increased in mice in response to ovariectomy. The mRNA expression of ALP and OCN and the ratio RANKL/OPG in mice femur were significantly reduced in either genistein- or 8PG-treated OVX mice (*P* < 0.05, vs OVX). In addition, treatment of OVX mice with 8PG at both doses dramatically reduced the mRNA expression of CtsK (*P* < 0.01) and high dose of 8PG markedly suppressed ovariectomy-induced expression of COL (*P* < 0.05) in femur of OVX mice (vs OVX). In contrast, genistein did not alter CtsK and COL mRNA expression in femur of OVX mice. Most importantly, treatment of OVX mice with genistein further increased the mRNA expression of ER-α (*P* < 0.05) while treatment with 8PG at both doses suppressed ER-α expression induced by ovariectomy (*P* < 0.05) in femur (vs OVX). The expression of GPER mRNA in mice femur was not altered in response to ovariectomy and different treatments.

Immunostaining (Figure 4E) showed that ER-α protein expression at distal femoral metaphysis in OVX mice was higher than that in Sham mice, and the treatment with genistein further increased the protein expression of ER-α of OVX mice. While, 8PG treatment at both doses reduced the ER-α expression at the metaphysis of distal femur as compared to that of OVX mice.

### Uterus index and estrogen-sensitive target expression

**Figure 5:**
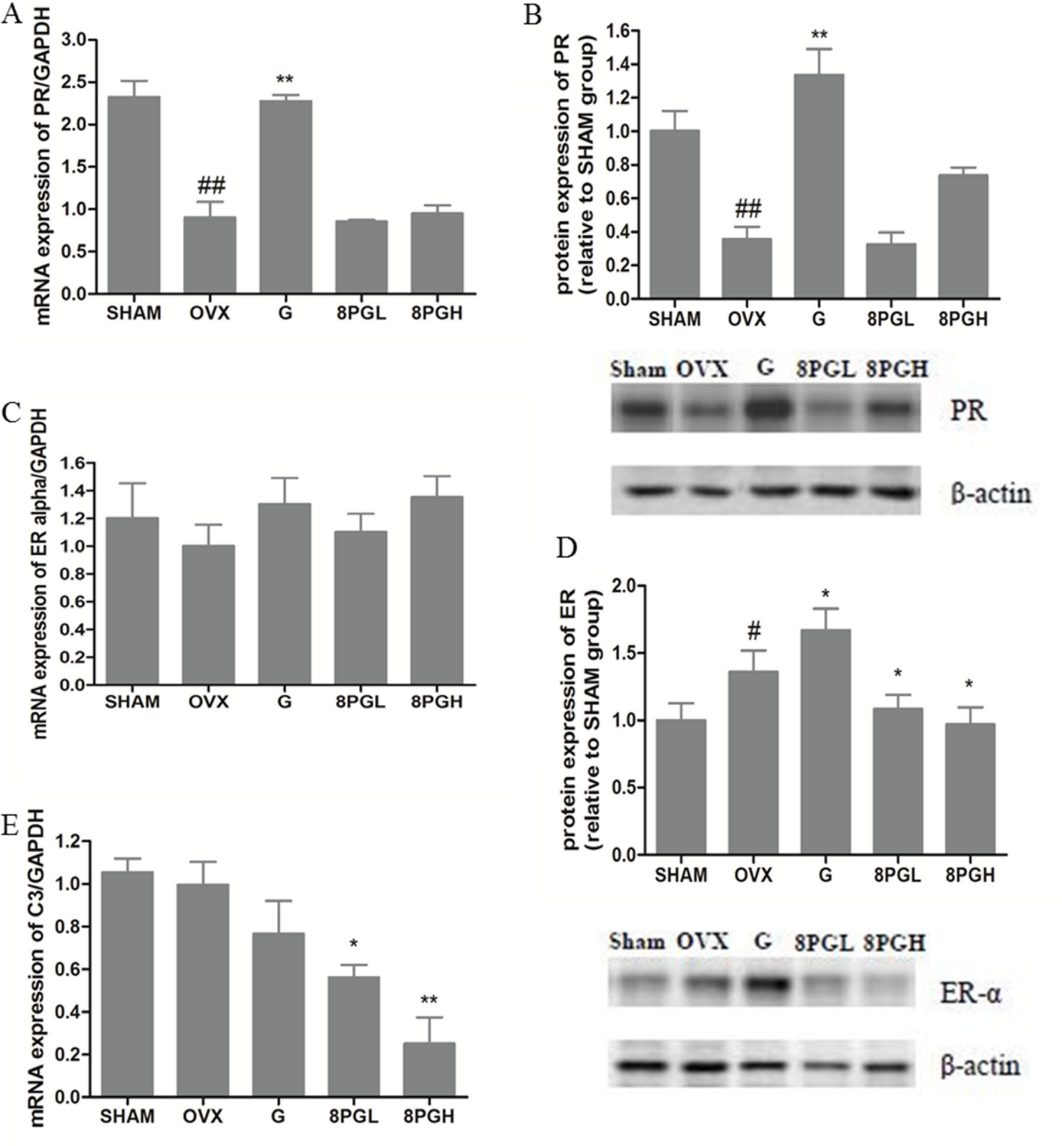
mRNA (**A**, **C**) and protein (**B**, **D**) expression of progesterone receptor (PR, A&B) and estrogen receptor-alpha (ER-α, C&D) as well as complement component 3 (C3) mRNA expression (**E**) in uterus of Sham mice and OVX mice treated with vehicle (OVX), genistein (G) or low (8PGL) or high (8PGH) dose of 8-prenylgenistein for 7 weeks. Values were expressed as means ± SEM, *n* = 10. ^#^*P* < 0.05, ^##^*P* < 0.01, vs. Sham group; ^*^P < 0.05, ^**^P < 0.01, vs. OVX group.

To evaluate the potential estrogenic effects of 8PG on uterus in estrogen-deficient mice, the uterus index and the expression of estrogen-targeting genes including ER-α, progesterone receptor (PR) and complement component 3 (C3) in uterus were measured. As expected, estrogen deficiency induced by ovariectomy resulted in the atrophy of uterus, as reflected by the significant decrease of uterus index in OVX mice (Table 1, *P* < 0.001, vs Sham). Treatment of OVX mice with genistein, but not 8PG at both doses, induced the increase of uterus index (*P* < 0.001, vs OVX). Our study also showed that genistein significantly increased the mRNA (Figure 5A) and protein (Figure 5B) expression of PR (*P* < 0.01) in OVX mice and further up-regulated the ovariectomy-induced increase of ER-α protein expression (Figure 5D, *P* < 0.05) in uterus of OVX mice (vs OVX). In contrast, PR expression and ER-α mRNA expression in uterus were not altered in OVX mice in response to treatment with 8PG (Figure 5A–5C), while ER-α protein expression in uterus was found to be suppressed in mice in response to 8PG treatment (Figure 5D, *P* < 0.05, vs OVX). In addition, mRNA expression of C3 was significantly decreased in uterus of OVX mice upon treatment with low dose (Figure 5E, *P* < 0.05) and high dose (*P* < 0.01) of 8PG treatment (vs OVX).

### Human endometrial Ishikawa cell

**Figure 6:**
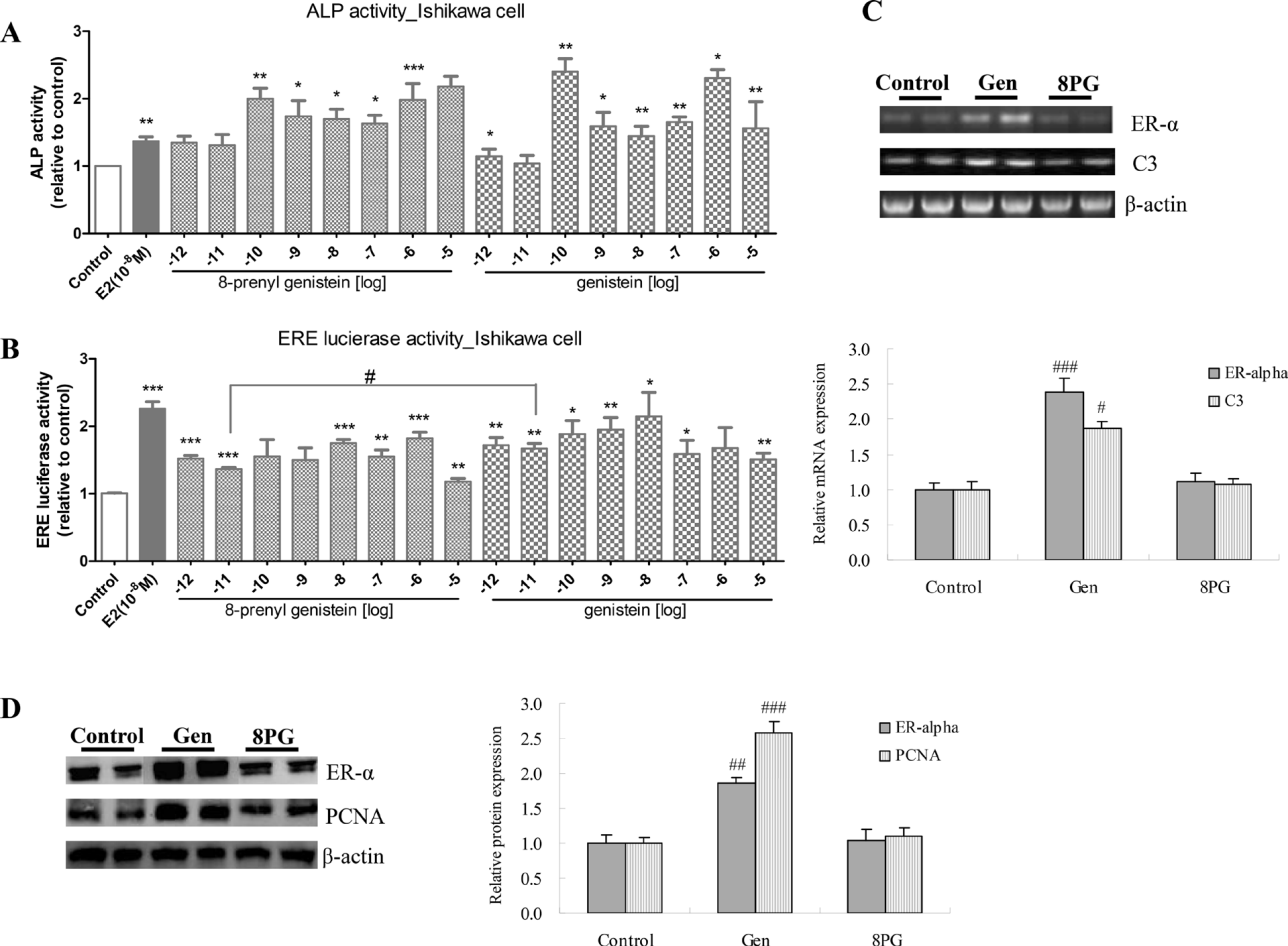
Alkaline phosphatase activity (ALP, **A**), estrogen response element (ERE)-dependent transcriptional activity (**B**), mRNA expression of ER-α and C3 (**C**) as well as protein expression of ER-α and proliferating cell nuclear antigen (PCNA) (**D**) in human endometrial Ishikawa cell. ALP and ERE activities were measured in Ishikawa cells upon to the treatment with vehicle (Control), 17-β estradiol (E2, 10^–8^ M), and 8-prenylgenistein and genistein at the concentration from 10^–12^ M to 10^–5^ M. Molecular expression was detected in Ishikawa cells treated with vehicle (Control), genistein (Gen, 10^–6^ M) and 8-prenylgenistein (8PG, 10^–6^ M) for 48 h. Values were expressed as means ± SEM. ^*^P < 0.05, ^**^P < 0.01, ^***^P < 0.001, vs. Control. ^#^*P* < 0.05, ^##^*P* < 0.01, ^###^*P* < 0.001, vs. Control.

To determine if 8PG exert direct estrogenic effects in endometrial cells, its effects on ALP activity, estrogen response element (ERE)-dependent transcriptional activities and ER-α expression in ER positive Ishikawa cells were determined. Moreover, the mRNA expression of C3 and the protein expression of proliferating cell nuclear antigen (PCNA), reflecting the proliferation of Ishikawa cells, were also measured. Both 8PG and genistein at the concentration from 10^–10^ M to 10^–6^ M stimulated ALP activity of Ishikawa cells (Figure 6A), and both of them stimulated the ERE activities in Ishikawa cells (Figure 6B), while, there were poor dose-dependent effects of 8PG and genistein on ALP activity and ERE activity. Genistein significantly increased mRNA (Figure 6C, *P* < 0.001) and protein (Figure 6D, *P* < 0.01) expression of ER-α associated with the up-regulation of C3 mRNA expression (Figure 6C, *P* < 0.05) and PCNA protein expression (Figure 6D, *P* < 0.001). The expression of ER-α, C3 and PCNA was not different between 8PG-treated group and control group.

### Binding affinity to estrogen receptor

**Figure 7:**
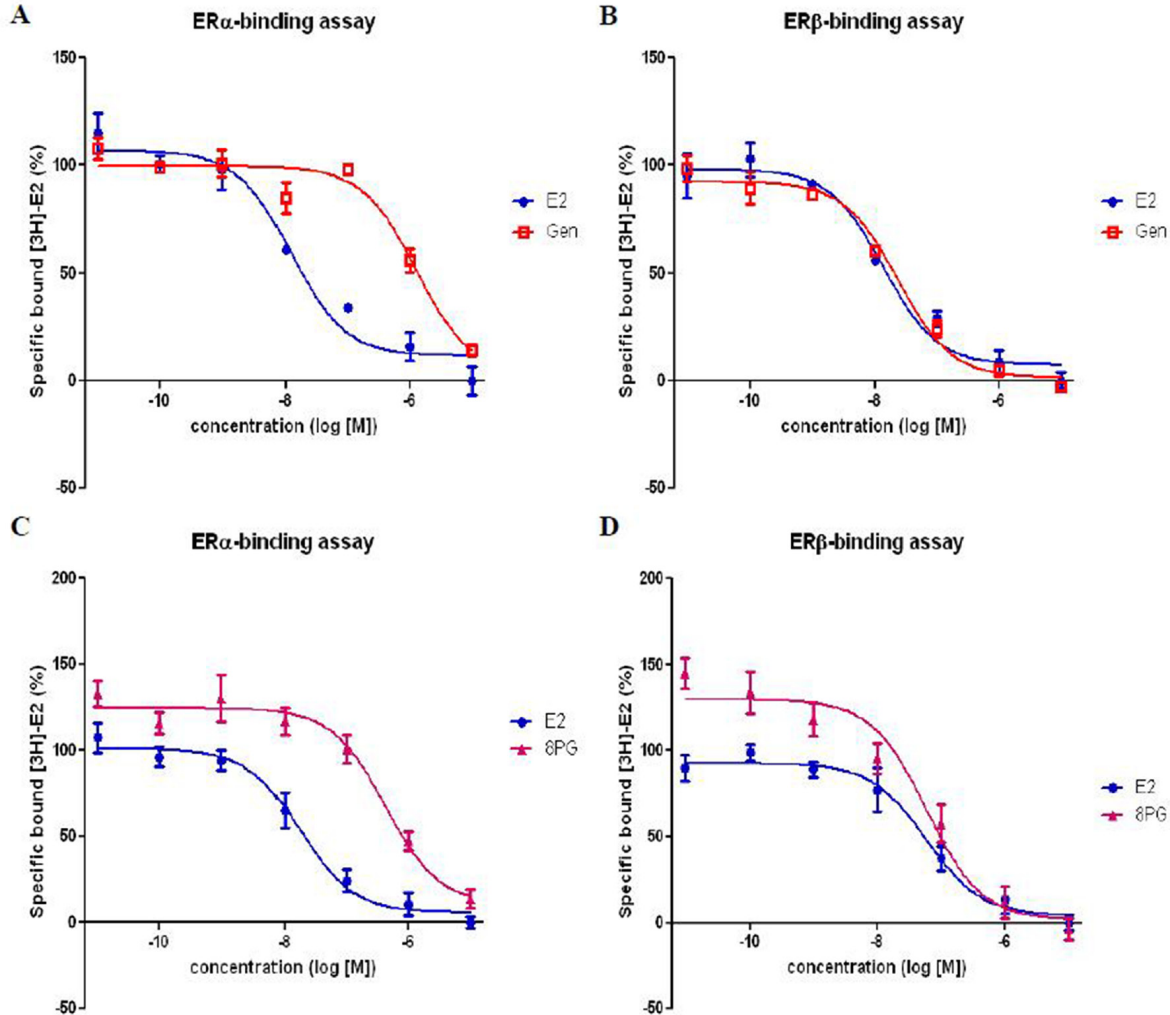
Competitive by genistein and 8-prenylgenistein for [H^3^]-E_2_ binding to recombinant human ER-α (**A**, **C**) and ER-β (**B**, **D**). Competition curves were obtained by adding increasing concentration of competitors to measure the displacement of bound radioligand. Results were expressed as percentage specific bound [H^3^]-E_2_ and obtained from three independent experiment. E2, 17-β estradiol; Gen, genistein; 8PG, 8-prenylgenistein.

As shown in Figure 7, increasing concentrations of 17-β estradiol (E2), genistein and 8PG decreased specific binding of [^3^H]-labeled E2 to recombinant ER-α (A & C) and ER-β (B & D). The results indicated that 8PG was a specific ligand to both ER-α and ER-β. As reported by others [27], the binding affinity of genistein towards ER-β (relative binding affinity (RBA) = 58.1%, Figure 7B) was stronger than towards ER-α (RBA = 0.99%, Figure 7A). The binding affinity of 8PG towards ER-α (RBA = 4.31%) was comparable to genistein but its binding affinity to ER-β (RBA = 99.6%, Figure 7D) was much higher than genistein.

### ERE-luciferase and ALP activities in MG63 cells

**Figure 8:**
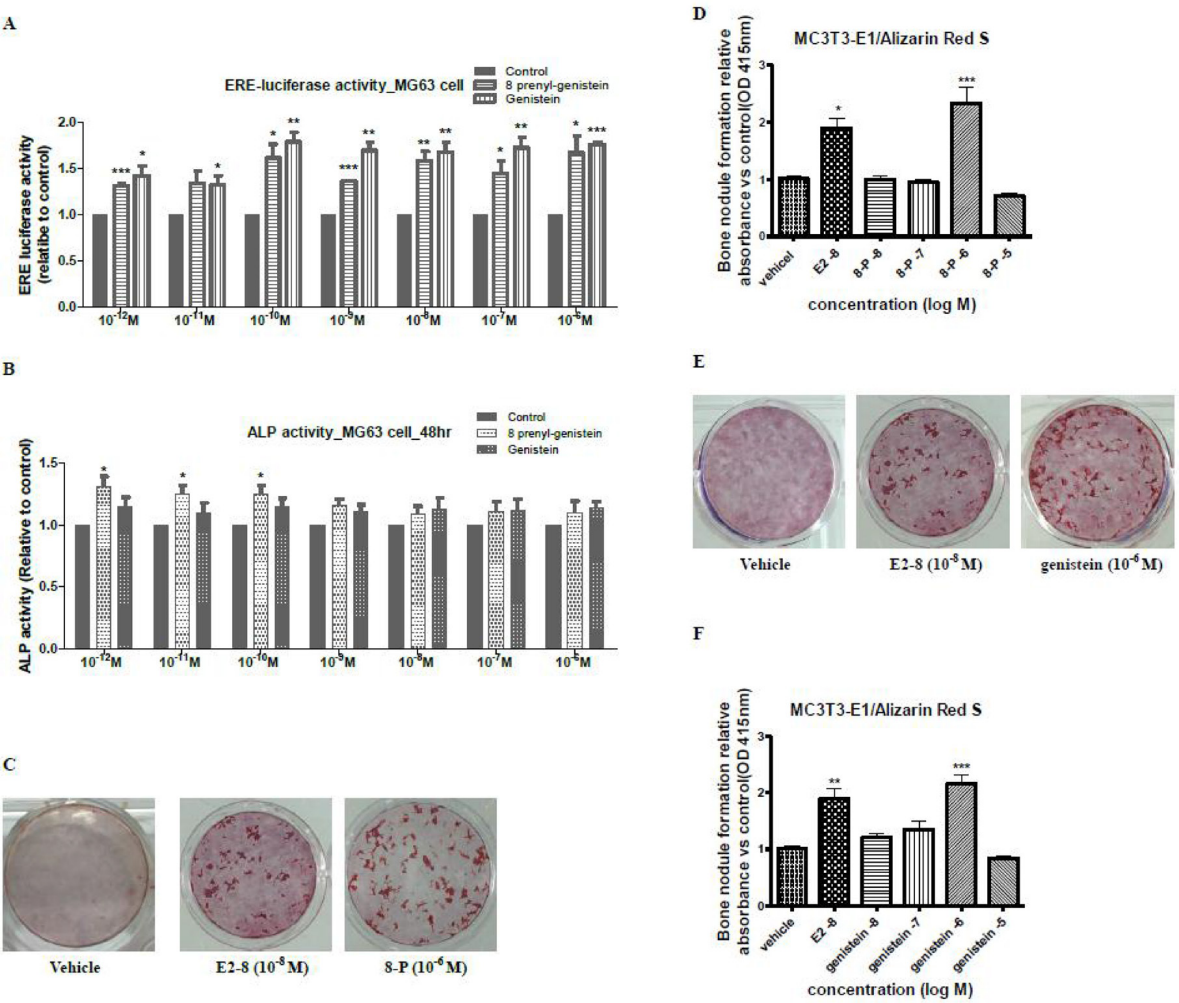
Estrogen response element (ERE)-dependent luciferase activity and alkaline phosphatase (ALP) activity of MG63 cells and bone nodule formation in murine osteoblastic MC3T3-E1 cells Activities of ERE-luciferase (**A**) and ALP (**B**) were measured in MG63 cells after treatment with genistein and 8-prenylgenistein at the concentration from 10^–12^ M to 10^–^^6^ M for 24 h and 48 h, respectively. Bone nodule formation by alizarin red staining was performed in MC3T3-E1 cells treated with vehicle, 17-β estradiol (E2, 10^–^^8^ M) and 8-prenylgenistein (**C**, **D**) and genistein (**E, F**) at the concentration from 10^–8^ M to 10^–^^5^ M for 21 days. Data were expressed as mean ± SEM. ^*^P < 0.05, ^**^P < 0.01, ^***^ *P* < 0.001, vs. Vehicle (Control) group.

To determine if 8PG and genistein transcriptionally induce estrogenic responses in bone cells, their abilities to induce ERE-dependent transcriptional activities in human osteosarcoma cell line MG63 cells were determined. MG63 cell transfected with ERE-luciferase construct were treated with vehicle control, genistein and 8PG at the concentration of 10^–12^~10^–6^ M (Figure 8A). Genistein and 8PG at all tested concentrations significantly increased ERE-dependent luciferase activity of MG63 cells. The ERE activities induced by genistein and 8PG in MG63 cells were comparable at most concentrations and no statistical difference was found between these treatments. 8PG significantly (*P* < 0.05) enhanced ALP activity of MG63 cells at the concentration of 10^–12^~10^–10^ M, however, genistein at tested concentration (10^–12^~10^–6^ M) did not alter ALP activity of MG63 cells (Figure 8B).

### Murine osteoblastic MC3T3-E1 cells

To further identify if 8-prenylgenistein and genistein differentially regulate the osteoblastic function, the bone nodule formation was determined in murine osteoblastic MC3T3-E1 cells (Figure 8C–8F). Calcium nodule was detected by alizarin red staining in MC3T3-E1 cells upon treatment with 10^–8^~10^–5^ M of 8PG and genistein. The most marked effect of 8PG and genistein on promoting nodule formation was observed at 10^–6^ M (*P* < 0.001) in which more than one fold increase in the amount of nodule formation were found (vs. vehicle control, Figure 8D and 8F). While, there was no dose-dependent effect for either 8PG or genistein under the tested concentration on calcium nodule formation.

## DISCUSSION

Our earlier structure-activity relationship study revealed that 8-prenylgenistein (8PG), a compound with prenylation at site 8 of ring A in parent structure of genistein, was more potent than genistein in increasing osteoblastic functions (proliferation, differentiation and mineralization) in osteoblasts-like cells UMR106 cells [22]. The present study further provided evidences that 8PG exhibited bone protective effects in ovariectomized (OVX) mice fed with phytoestrogen-free diet. Moreover, unlike genistein, 8PG did not induce uterotrophic effects in OVX mice nor change the expression of ERα and proliferating cell nuclear antigen (PCNA) in Ishikawa cells. It is of particular interest to note that the regulation of ERα expression in bone and uterus of OVX mice by 8PG and genistein was different in which 8PG suppressed and genistein induced its expression in both tissues, suggesting that 8PG exerted tissue-selective estrogen-like effects in bone, but not uterus, in OVX mice.

Our study clearly demonstrated that 8PG exerted protective effects against bone loss in mice induced by estrogen deficiency. The results also showed that 8PG dose-dependently suppressed ovariectomy-induced elevation of urinary calcium excretion. 8PG significantly suppressed ovariectomy-induced increase in serum osteopontin levels, a bone resorption marker in mice [28, 29]. Micro-CT analysis indicated that 8PG treatment significantly improved trabecular bone mineral density and micro-architecture at femoral end and tibial head in OVX mice. The histomorphological staining and reconstructed 3-dimensional images consistently showed that 8PG improved trabecular bone network and increased trabecular bone mass at distal metaphysis of femur and proximal metaphysis of tibia as well as improved the histological structure of articular cartilage at tibial epiphysis. These results fully showed that 8PG was effective in repressing the bone deteriorations induced by estrogen deficiency in OVX mice.

The responses of mRNA expression of genes involved in bone metabolism in OVX mice were studied in the present study in an attempt to characterize the differential actions of 8PG and genistein on bone. Our results clearly showed that the expression of genes involved in bone formation (ALP, OCN, COL) and gene involved in bone resorption (CtsK) in femur were significantly induced in OVX mice, a typical feature of high bone turnover associated with estrogen deficiency. Treatment of OVX mice with genistein significantly down-regulated the mRNA expression of ALP and OCN and reduced the ratio of RANKL/OPG (a marker for osteoclastogenesis) expression [30]. Similar results were reported by others that genistein reduced ALP activity and the expression of COL and OCN in human bone marrow mesenchymal stem cells [31] and suppressed the ratio of RANKL/OPG expression in bone of OVX mice [32]. Most importantly, our results showed that 8PG at both doses not only suppressed the expression of genes involved in bone formation (ALP, OCN) and osteoclastogenesis (RANKL/OPG), but also suppressed the expression of gene involved in bone resorption (CtsK) in OVX mice. Indeed, the suppression in Ctsk mRNA expression by 8PG was associated with the suppression of bone resorption marker in OVX mice, suggesting that 8PG might exert inhibitory actions on the process of bone resorption. Thus, our results clearly indicated that 8PG and genistein exerted differential actions on bone and that the suppression of the process of bone resorption by 8PG might account for its potent actions in protecting against rapid bone turnover induced by estrogen deficiency in mice.

One of the major concerns regarding the use of phytoestrogens, like genistein, was the undesirable effect in other reproductive organs [7, 8]. To evaluate if 8PG exerted estrogenic effects in the reproductive tissues, the uterotrophic responses in OVX mice fed genistein and 8PG were compared. Treatment with genistein for 7 weeks markedly increased the uterus index in OVX mice. Moreover, genistein significantly increased PR (a gene regulated by estrogen) mRNA and protein expression as well as ERa protein expression in uterus in OVX mice. In contrast, 8PG at both low and high dose did not increase uterus index nor increase PR mRNA and protein expression in uterus in OVX mice. Furthermore, 8PG reduced both ERa protein expression and C3 (another estrogen-sensitive gene) mRNA expression in uterus in OVX mice. Such antagonism of estrogenic effects has also been reported for dihydrotestosterone (DHT), in which ERa expression in the endometrial stroma in OVX pigs was shown to be down-regulated by DHT [33]. These results clearly indicated that the effects of 8PG and genistein in uterus of OVX mice were different.

To further evaluate their potential estrogenic activities in human endometrial cells [34], their abilities to induce the activity of ALP and expression of PCNA, C3 and ER-α in Ishikawa cell were studied. PCNA is usually used as a biochemical marker for evaluating the proliferation of endometrial cancer cells [35], and C3 is one of the estrogen-responsive genes [36]. PCNA expression was induced by estrogen in breast cancer MCF-7 [37] and by genistein in a time- (at 48 h and afterward) and concentration-dependent manner (at 10(-8) M and above) in Ishikawa cells [38]. Our results clearly indicated that genistein (1 μM), but not 8PG (1 μM), induced ER-α, PCNA and C3 expression in Ishikawa cells. Indeed, the observations were similar to those reported by Kretzschmar et al. [17] in which genistein was much more potent than 8PG in inducing estrogenic responses in yeast based assay. Indeed, the results of our *in vitro* study were in agreement with those in the *in vivo* study that 8PG and genistein exerted differential actions in endometrial cells and uterus. Our results indicated that 8PG, with modification by prenylation in the chemical structure of genistein, diminished its side effects on reproductive tissues and exhibited tissue-selective estrogen-like effects on bone.

It is well known that in the classical pathway the actions of estrogen at cellular levels are mediated through ERs, mainly include ERα and ERβ, and subsequently alter gene transcription [39]. Moreover, ERα is reported to be the main subtype of ERs that is involved in key biological actions in bone [40, 41] and uterus [42, 43]. Our study demonstrated that the affinity of genistein and 8PG to ERα was comparable and weaker than estrogens. Our results also showed that the abilities of 8PG and genistein to activate ERE-dependent transcriptional activities in human osteoscaroma MG63 cells and human endometrial Ishikawa cell, and to stimulate ALP activity, an estrogenic response marker, in Ishikawa cells and in MG63 cells were comparable at most of concentration tested. Thus, the differential actions of 8PG and genistein in bone and uterus were not likely due to the difference in ERα binding affinity nor their abilities to induce ERE-dependent transcriptional or ALP activities in these tissues.

Interestingly, it appeared that the decrease in ERα expression in uterus might account for the decrease in uterotrophic responses (uterus index and C3 expression) in 8PG-treated OVX mice. Whereas, the decreased expression of ERα in bone tissue by 8PG may not account for its observed bone protective effects in OVX mice, as a decrease in estrogenic effects in bone means a decrease in anabolic effects on bone and an increase in bone resorption. Thus, our study suggested that other non-ERα dependent pathways, like Wnt/β-catenin signaling and BMP signaling, might be involved in protective effects of 8PG on bone.

The key characteristics of 8PG in comparison to genistein were summarized as shown in Table 3. In conclusion, the present study revealed the osteoprotective effects of 8-prenylgenistein without uterotrophic action in OVX mice. The tissue selective effect of 8PG may, at least partially, be accounted for its modulatory effects on ERα expression in bone and uterus. Recent research studies indicated that the actions of estrogen can also be mediated by rapid signaling events that involved transmembrane ER [44]. Future study will be needed to delineate the other mechanisms, like nongenomic rapid signaling pathway and non-ERα dependent pathway, by which 8-prenylation achieves *in vivo* tissue selectivity.

**Table 3:** Differential actions of genistein and 8-prenylgenistein on bone and uterus in ovariectomized mice and human endometrial Ishikawa cell

	Genistein	8-prenylgenistein
**Biochemical markers** Serum Ca Urinary Ca Bone resorption marker (serum OPN)	No change No change No change	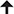 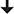 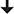
**Bone properties** Tibial head BMD Tb.Th DA Tb.Sp	No change No change No change No change	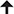 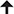 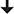 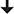
Femoral end BMD BV/TV Tb.N Tb.Th Tb.Sp	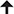 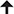 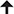 No change 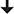	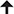 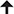 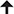 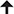 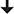
**Uterus response** Uterus index PR ER-α C3	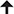 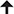 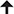 No change	No change No change 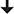
Ishikawa cell ER-α PCNA C3		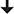 No change No change No change
**Binding affinities (RBA)** ER-α ER-β	0.99% 58.1%	4.31% 99.6%

BMD, bone mineral density; BV/TV, bone volume over total volume; C3, complement component 3; Ca, calcium; DA, degree of anisotropy; ER-α, estrogen receptor alpha; ER-β, estrogen receptor beta; OPN, osteopontin; PCNA, proliferating cell nuclear antigen; PR, progesterone receptor; RBA, relative binding affinity; Tb.N, trabecular bone number; Tb.Sp, trabecular bone separation; Tb.Th, trabecular bone thickness.

## MATERIALS AND METHODS

### Compounds

Genistein was obtained from Sigma, and 8-prenylgenistein (8PG) was synthesized analogously starting from genistein (Changzhou University, Jiangsu, China). The purity of the used compounds was assessed to be > 99% (gas chromatography and high-performance liquid chromatography).

### Animals study design

Three-month-old female C57BL/6J mice (The Chinese University of Hong Kong) were housed at the Centralized Animal Facility of the Hong Kong Polytechnic University with alternating 12 h periods of light and darkness, a constant temperature of 23 ± 1°C, and humidity of 55 ± 5% upon arrival. The mice were acclimated for 1 month during which they were allowed free access to deionized distilled water and phytoestrogen-free diet (D00031602, Research Diet Inc, New Brunswick, NJ, USA). The latter was used as the control diet in this study and was prepared according to the AIN-93M formulation where corn oil was used instead of soybean oil. Soy oil was substituted by corn oil as a fat source in order to prevent any additional components of soy from being added to the diets. The mice were either dorsal ovariectomized (OVX) or sham-operated (Sham) under light ether anesthesia. Starting from post-surgery, the mice were divided into five groups: Sham-operated mice with control diet (Sham, *n* = 10), OVX mice with control diet (OVX, *n* = 10), OVX mice with genistein (D13052408, 600 mg/kg diet, *n* = 10) or low dose (D13052402, 310 mg/kg diet, *n* = 10, 8PG-L) or high dose (D13052402, 620 mg/kg diet, *n* = 10, 8PG-H) of 8-prenylgenistein. The diets were pelleted by dry extrusion and color-coded. The nutritional composition of different diets was shown in Supplementary File (Supplementary Table 1). All mice in each group were individually caged and pair-fed with approximately 5 g/day of the respective diet which was completely consumed by each animal. After 7 weeks of treatment, urine was collected by using metabolic cage. All mice were killed by cardiac exsanguination and then serum was prepared and stored at –80°C for further biochemical analyses. The uterus, the bilateral tibias and femurs were aseptically removed and stored. All procedures were reviewed and approved by the Animal Ethics Committee of The Hong Kong Polytechnic University (approved No: 12/35).

### Chemistries in serum and urine

Calcium (Ca) and phosphorus (P) concentrations in serum and urine were measured by standard colorimetric methods. The content of osteocalcin (Immutopics, San Clemente, USA) and osteopontin (ALPCO, Salem, USA) in serum were assayed by using commercial ELISA kits.

### Micro-computed tomography (Micro-CT) analysis

The proximal metaphysis of tibia and the distal end of femur were scanned with a high-resolution micro viva CT 40 system (Scanco Medical, Bassersdorf, Switzerland). Trabecular bone was determined by a fixed threshold. The scans resulted in reconstructed 3-dimensional (3-D) data sets with μCT Evaluation Program (V5.0A). After images were captured, 100 slices were established as the volume of interest. Trabecular bone was separated from cortical bone by free drawing regions of interests using the software provided with the scanner. Morphologic measurements of the metaphyseal trabecular bone for the 100 slices were performed to obtain 3-D images and the quantitative parameters as the following: (1) the mean bone mineral density of total volume (BMD/TV); (2) connectivity density (Conn.D); (3) bone volume (BV)/TV; (4) the geometric degree of anisotropy (DA); (5) trabecular bone number (Tb.N); (6) trabecular bone separation (Tb.Sp); (7) trabecular bone thickness (Tb.Th).

### Histological and immunohistochemical staining on trabecular bone

The tibias and femurs were fixed, decalcified, and embedded in paraffin by standard histological procedures. Serial sections of 3 μm were cut. Safranin O (Sigma-Aldrich) staining was performed, combining with fast green and counter stain by hematoxylin. Additionally, the alcian blue/hematoxylin & orange G staining was performed on articular cartilage of tibial head. The protein expression of estrogen receptor-alpha (ER-α) at distal metaphysis of femur was detected by immunohistochemical staining. Stained slides were visualized under microscope.

### RT-PCR and quantitative RT-PCR

RNA from the distal one third of the whole femur of each animal was protected from degradation by liquid nitrogen when being grinded in a mortar. Bone powder was put in an eppendorf tube filled with TRIzol reagent; Uterus was thawed in TRIzol reagent and homogenized. Total tissue RNAs was isolated according to the TRIzol manufacturer's protocol (Invitrogen, Carlsbad, California, USA). Synthesis of cDNAs was performed by reverse transcription reactions using moloney murine leukemia virus reverse transcriptase (Invitrogen, Carlsbad, California, USA). The first strand cDNAs served as the template for the regular PCR performed using a DNA Engine (ABI). Real-time RT-PCR was performed with the Applied Biosystems 7900 Real Time PCR System using a SYBR green PCR reagent kit (Applied Biosystems, Foster City, CA). Glyceraldehyde-3-phosphate dehydrogenase (GAPDH) as an internal control was used to normalize the data to determine the relative expression of the target genes, including osteoprotegerin (OPG), receptor activator of nuclear factor kappa-B ligand (RANKL), ER-α, G protein-coupled estrogen receptor (GPER), alkaline phosphatase (ALP), type I collagen (COL), osteocalcin (OCN), cathepsin K (CtsK), progesterone receptor (PR), complement component 3 (C3). The PCR primers used in this study were shown in Supplementary File (Supplementary Table 2).

### Immunoblotting

Uterus was thawed in Laemmli buffer (Boston Bioproducts, Worcester, MA, USA) and homogenized for protein isolation. The cell proteins were also extracted in Laemmli buffer. Protein samples were separated on SDS-PAGE gel, transferred to PVDF membranes (Whatman). After saturation with 5% (w/v) nonfat dry milk in TBS and 0.1% (w/v) Tween 20 (TBST), the membranes were incubated with rabbit polyclonal IgG ER-α, PR or proliferating cell nuclear antigen (PCNA) and followed by incubation with goat anti-rabbit conjugated with horseradish peroxidase. The antigen-antibody complexes were then detected with enhanced chemiluminescence (ECL) reagent and visualized by the Lumi-Imager using Lumi-Analyst version 3.10 software (Roche, Mannheim, Germany).

### Human endometrial Ishikawa cell

An immortalized human uterine endometrial adenocarcinoma cell line (Ishikawa) was obtained from ATCC (American Type Culture Collection, Manassas, USA). Cells were grown in a standard tissue culture incubator at 37°C, with 95% humidity and 5% carbon dioxide. Ishikawa cells were maintained in DMEM/F12 (Hyclone, USA) supplemented with 10% FBS (Gibico, USA). Cells were seeded in a 6-well plate, and twenty-four hours prior to cell treatment, media were changed to phenol red-free DMEM/F12 containing 5% charcoal-stripped heat-inactivated FBS. Cells were treated with genistein (10^–6^ M) or 8-prenylgenistein (10^–6^ M) dissolved in ethyl alcohol absolute (EtOH). EtOH diluted in medium was used as blank control. Upon treatment for 48 h, RNA was isolated by TRIzol and protein was obtained by RIPA lysis buffer supplemented with proteinase inhibitors cocktail (Roche, Mannheim, Germany). RT-PCR and immunoblotting for ER-α, C3 and proliferating cell nuclear antigen (PCNA) were further performed.

### Binding affinity assay with purified ERα and ERβ

96-well microtiter filter plates (Millipore, USA) were used in the assay. Each compound was diluted with 1:1 dimethylsulf- oxide assay buffer (50 mM Tris, 10% glycerol, pH 8.0, 0.3 mg/ml ovalbumin, 0.01 M mercaptoethanol) to seven concentrations from 7 × 10^–11^ M to 7 × 10^–5^ M. 10 μl compound dilutions, 10 μl ^3^[H]E2 (7 × 10^–8^ M) and 50 μl recombinant ER protein (1.0 × 10^–9^ M) (PanVera/Invitrogen Corp, Carlsbad, USA) were loaded into each well. The plates were shaken in an orbital shaker for 5 min and incubated overnight (18–24 h) on ice. HAP slurry (Bio- Rad Pacific Ltd., USA) was added and incubated for 15 min at 0°C to capture the protein. The trapped proteins were washed twice with ice cold HAP washing buffer and re-suspended in HAP washing buffer. Portions of the solution were mixed with scintillation fluid (Fisher Scientific, USA) and subjected to measurement by a liquid scintillation counter (Beckman LS6500 Scintillation Counter, USA). The radioactivity of each sample was expressed as disintegration per minute (dpm). The binding of ^3^[H]E2 to ER in the presence of competitor was determined by subtracting the non-specific binding and expressed as percentage of total binding without competitor. The relative binding affinities (RBA) were calculated by (IC_50_ 17β-estradiol/IC_50_ compound) × 100.

### Alkaline phosphatase (ALP) activity

Ishikawa cells or the human osteosarcoma cells (MG-63) were seeded and cultured in 24-well plate at the density of 5 × 10^4^/well. 24 hours later, the medium was changed to phenol-red free MEM containing 2% charcoal stripped FBS (cs-FBS) for another 24 h followed by treatment with 8PG or genistein at different concentrations for 48 h. Cells were rinsed by PBS for three times and lysed with 100 μl of 1× passive lysis buffer (PLB, Promega) per well overnight at –20°C. ALP activity of each well was measured by LabAssayTM ALP kit (Wako) according to the manufacturer's instruction. Protein concentration of treated cells was measured by Bradford assay to normalize the ALP activity.

### Estrogen response element luciferase activity

MG63 cells and Ishikawa cells were seeded in 24-well plate at the density of 3 × 10^4^/well. Cells were transfected with 0.4 μg pERE-luc and 0.01 mg pRL-TK with lipofectamine 2000 and incubated for 6 h. The cells were treated with vehicle, various concentrations of 8-prenylgenistein and genistein for 24 h. Firefly and Renilla Luciferase activities were measured sequentially with Dual Luciferase Assay reagents.

### Bone nodule formation of murine osteoblastic MC3T3-E1 cells

MC3T3-E1 cells were seeded in 12-well plate at a density of 2 × 10^4^ cells/well. The medium was changed to differentiation medium (α-MEM with 10% FBS and 25 μg/ml of ascorbic acid and 10 mM of β-glycerophosphate) with 10^–8^ M E2 (positive control), vehicle (1% ethanol) and different concentrations of 8PG and genistein (10^–8^ M to 10^–5^ M). The medium was changed every 2~3 days. After 21 days, the cells were rinsed with PBS for two times and fixed in 4% formaldehyde. Cells were then stained with 1% Alizarin Red for 10 minutes and captured under light microscope by using camera (Olympus, Japan). In order to quantify the bone nodule formation, 0.5 M HCl and 5% SDS were added to the stained wells, which were shook for 30 minutes to completely dissolve cells. The optical density was measured at 415 nm by spectrophotometric plate reader (Bio-Rad model 550, Japan).

### Statistical analysis

The data from these experiments were reported as mean ± standard error of mean (SEM) for each group. All statistical analyses were performed using PRISM version 4.0 (GraphPad). Inter-group differences were analyzed by one-way ANOVA, and followed by Tukey's multiple comparison test as a post test to compare the group means if overall *P* < 0.05. Differences with *P* value of less than 0.05 were considered statistically significant.

## SUPPLEMENTARY MATERIALS TABLES


