# Supplementary material for: 8-Prenylgenistein, a prenylated genistein derivative, exerted tissue selective osteoprotective effects in ovariectomized mice
Source: Oncotarget. 2018 Mar 19;9(36):24221–36. doi: 10.18632/oncotarget.24823 (PMC5966244; doi:10.18632/oncotarget.24823)
Supplement: Supplementary file 1 [file oncotarget-09-24221-s001.pdf]

8-Prenylgenistein, a prenylated genistein derivative, exerted tissue selective osteoprotective effects in ovariectomized mice

SUPPLEMENTARY MATERIALS

Supplementary Table 1: Formulations and estimated nutrient composition of experimental diets

Product[1]	D00031602		D13052402		D13052403		D13052408	
	gm%	kcal%	gm%	kcal%	gm%	kcal%	gm%	kcal%
Protein	14.2	14.7	14.2	14.7	14.2	14.7	14.2	14.7
Carbohydrate	73.1	75.9	73.0	75.9	73.0	75.9	73.0	75.9
Fat	4.0	9.4	4.0	9.4	4.0	9.4	4.0	9.4
Total		100.0		100.0		100.0		100.0
kcal/gm	3.85		3.85		3.85		3.85	
**Ingredient**	***gm***	***kcal***	***gm***	***kcal***	***gm***	***kcal***	***gm***	***kcal***
Casein	140	560	140	560	140	560	140	560
L-Cystine	1.8	7.2	1.8	7.2	1.8	7.2	1.8	7.2
Corn Starch	495.692	1982.8	495.692	1982.8	495.692	1982.8	495.692	1982.8
Maltodextrin 10	125	500	125	500	125	500	125	500
Sucrose	100	400	100	400	100	400	100	400
Cellulose, BW200	50	0	50	0	50	0	50	0
Corn Oil	40	360	40	360	40	360	40	360
t-Butylhydroquinone	0.008	0	0.008	0	0.008	0	0.008	0
Mineral Mix S10022M2	35	0	35	0	35	0	35	0
Vitamin Mix V100373	10	40	10	40	10	40	10	40
Choline Bitartrate	2.5	0	2.5	0	2.5	0	2.5	0
8-prenylgenistein	0	0	0.31	0	0.62	0	0	0
Genistein	0	0	0	0	0	0	0.6	0
Red Dye, FD&C #40	0.1	0	0.025	0	0	0	0	0
Blue Dye, FD&C #1	0	0	0	0	0.05	0	0.01	0
Yellow Dye, FD&C #5	0	0	0.025	0	0	0	0.04	0
**Total**	**1000.1**	**3850**	**1000.36**	**3850**	**1000.67**	**3850**	**1000.65**	**3850**

[1]Prepared by Research diets, Inc., New Brunswick, NJ.
[2]The mineral mix composition (AIN-93M) was as follows (amount in 35 g): 5.0 g Ca, 2.0 g P, 0.5 g Mg, 3.6 g K, 0.3 g S, 1.0 g Na, 1.6 g Cl, 6.0 mg Cu, 0.2 mg l, 45 mg Fe, 10.5 mg Mn, 0.2 mg Se and 30.0 mg Zn.
[3]The vitamin mixture composition (AIN-93) was as follows (amount in 10 g): 4000 IU vitamin A palmitate, 1000 IU cholecalciferol, 75 IU vitamin E acetate, 0.75 mg phylloquinone, 0.2 mg biotin, 25 µg cyanocobalamin, 2 mg folic acid, 30 mg nicotinic acid, 16 mg calcium pantothenate, 7 mg pyridoxine-HCl, 6 mg riboflavin, 6 mg thiamin HCl.

Supplementary Table 2: Primer sequences of genes

Gene (Abbreviation)	Full name	Primer sequences (5`-3`)	Product length (bp)
RANKL	Receptor activator of nuclear factor kappa-B ligand	F: gaaaggagggagcacgaaaa<br>R: tgaaagccccaaagtacgtc	167
OPG	Osteoprotegerin	F: ctgatgtatgccctcaagca<br>R: aaacagcccagtgaccattc	198
ER-α	Estrogen receptor-alpha	F: tcccgccttctacaggtcta<br>R: ctccggttcttgtcaatggt	264
GPER	G protein-coupled estrogen receptor	F: cctgggtagagggacattag<br>R: gctcgaagtgggaaatacag	112
RUNX2	Runt-related transcription factor 2	F: cccagccacctttacctaca<br>R: tatggagtgctgctggtctg	150
ALP	Alkaline phosphates	F: gaaagagaaagaccccagtt<br>R: ctcattgtccgagtaccagt	511
COL	Type I collagen	F: cagactggcaacctcaagaa<br>R: ggccaatgtctagtccgaat	241
OCN	Osteocalcin	F: gacaccatgaggaccatcttt<br>R: tagagaccactccagcaca	225
SOST	sclerostin	F: cttcaggaatgatgccacagaggt<br>R: atctttggcgtcatagggatggtg	136
CtsK	Cathepsin K	F: gagacatgaccagcgaagaa<br>R: cacatattggaaggcagtgg	332
PR	Progesterone receptor	F: ggtgggccttcctaacgag<br>R: gaccacatcaggctcaatgct	121
C3	Complement component 3	F: ggagccagtggacatctgaga<br>R: ccctccttatctgagtttgaattcct	74
GAPDH	Glyceraldehyde-3-phosphate dehydrogenase	F: cagaacatcatccctgcatc<br>R: ctgcttcaccaccttcttga	183
ER-α*	Estrogen receptor-alpha	F: cgtcgcctctaacctcgg<br>R: tcccagatgctttggtgtg	140
C3*	Complement component 3	F: gccaggagtggactatgtgt<br>R: ggcttctctgcacttgatgg	166

*, for human endometrial Ishikawa cell.
